# *Lactobacillus acidophilus* combined with *Pediococcus acidilactici* ameliorates colitis

**DOI:** 10.3389/fmicb.2026.1773197

**Published:** 2026-03-04

**Authors:** Peilin Yu, Yuanming Jin, Da-Jeong Park, Mingzhu Wang, Chong-Su Cho, Chunri Yan, Fuliang Sun, Xin Jin, Keesun Yu, Young Jin Pyung, Cheol-Heui Yun, Lianhua Cui

**Affiliations:** 1 College of Agriculture, Yanbian University, Yanji, China; 2 Engineering Research Center of North-East Cold Region Beef Cattle Science and Technology Innovation, Ministry of Education, Yanbian University, Yanji, China; 3 Department of Agricultural Biotechnology, Research Institute of Agriculture and Life Sciences, Seoul National University, Seoul, Republic of Korea; 4 Department of Preventive Medicine, Medical College, Yanbian University, Yanji, China; 5 Laboratory Animal Center, Yanbian University, Yanji, China; 6 Institutes of Green Bio Science and Technology, Seoul National University, Pyeongchang, Republic of Korea

**Keywords:** colitis, inflammatory response, intestinal integrity, *Lactobacillus acidophilus*, microbiota, *Pediococcus acidilactici*, probiotics

## Abstract

**Background:**

The increasing global incidence of ulcerative colitis (UC) calls for urgent attention to the prevention and management of its symptoms. Public awareness and international regulations aimed at banning or reducing antibiotic use require alternative strategies, with probiotics demonstrating promising potential. Recent studies suggest that the combination of different probiotic strains with complementary functions may achieve synergistic effects.

**Methods:**

We selected *Lactobacillus acidophilus*, noted for its mucosal adhesion, and *Pediococcus acidilactici*, distinguished for its environmental resilience, to investigate their combined effects on the alleviation of symptoms in a mouse model with DSS-induced colitis.

**Results:**

The results showed that the combined intervention was effective in reducing weight loss in mice with colitis and in mitigating the disease activity score. The combination significantly alleviated conditions such as colonic crypt dysfunction, goblet cell loss, and severe mucosal damage. Serum biochemical indicators revealed that the combined lactic acid bacteria increased the antioxidant capacity of the mice. Furthermore, administration of the combination reduced levels of inflammatory cytokines in colon tissues and increased the mRNA expression levels of tight junction proteins. It partially reversed changes in the gut microbiota in mice with colitis, mainly by increasing the abundance of potentially beneficial bacteria such as *Akkermansia*, together with increasing short-chain fatty acids production in the cecum.

**Discussion:**

The current study demonstrates that the combination of *Lactobacillus acidophilus* and *Pediococcus acidilactici* exerts protective effects against colitis in mice by the enhancement of antioxidant capacity, reduced inflammatory responses, preservation of intestinal barrier integrity, and partial restoration of gut microbiota and its metabolite production. Collectively, the study provides novel insights into the synergistic application of the specific probiotic pair for colitis management.

## 1 Introduction

Inflammatory Bowel Disease (IBD) comprises a group of chronic conditions characterized by recurrent inflammation of the gastrointestinal tract, with its two major clinical phenotypes being Ulcerative Colitis (UC) and Crohn’s Disease (CD) (Torres et al., 2017). Although the precise mechanisms underlying IBD remain unclear, accumulating evidence suggests that genetic susceptibility, environmental factors, and intestinal microbiota dysbiosis collectively contribute to disease development (Guan, 2019). Traditional treatments for UC include conventional therapies such as aminosalicylates, corticosteroids, immunosuppressants, and biologics. While these can induce remission, their efficacy is limited and they are often associated with significant side effects, high relapse rates, and financial burdens (Cai et al., 2021). Given the key role of gut microbial dysbiosis in the pathogenesis of IBD, antibiotics are sometimes used as adjunctive therapy to temporarily control inflammation (Xie et al., 2025). However, the misuse of antibiotics can not only induce bacterial resistance but also further disrupt the gut microbial balance, worsening disease recurrence and limiting long-term effectiveness (Nitzan et al., 2016). With the increasing global attention to antibiotic resistance (AMR) and stricter related regulations, along with the limitations of traditional immunosuppressive therapies, there is a growing research focus on finding safer, low-side-effect alternatives that can restore gut homeostasis (Dongre et al., 2025). Therefore, safe and side-effect-free microecological preparations have gained significant attention. Strategies such as probiotics, prebiotics, and synbiotics have been proven to effectively enhance gut health and immune function while reducing reliance on antibiotics (Iduu et al., 2025). Recent studies suggest that the multi-species probiotic combination was more effective than the species individually in repairing the dysbiosis of mucosal microbial ecology and reducing intestinal inflammation (Wang et al., 2019). This highlights the potential of multi-species preparations as promising alternative strategies for restoring intestinal homeostasis.

*Lactobacillus acidophilus* has been believed to adhere to intestinal epithelial cells, thereby forming a physical barrier that inhibits the colonization of pathogenic bacteria. Additionally, its adhesion factors facilitate interactions with host cells, contributing to the maintenance of intestinal mucosa integrity, enhancing pathogen clearance, and regulating host immune responses (Goh and Klaenhammer, 2010). *Pediococcus acidilactici* has attracted interest due to its potent physiological activity and flexibility (Dong et al., 2025). *P. acidilactici* has been proposed to consistently produce bacteriocins. Bacteriocin-producing strain can colonize and persist in the gut, modulating the micro-ecological structure in the host and promoting the proliferation of beneficial bacteria, while its bacteriocins simultaneously exert antimicrobial activity that inhibits pathogens with overlapping ecological niche and nutritional demands, thereby reinforcing colonization resistance (Parada et al., 2007). Additionally, its robust acid tolerance ensures a high survival rate in the digestive tract (Li et al., 2021). *P. acidilactici* can also regulate the host’s immune function by producing metabolites such as lactic acid and short-chain fatty acids (SCFAs) (Zhao et al., 2024). It has been shown that *L. acidophilus* and *P. acidilactici* exhibit inhibitory effects on intestinal pathogens, and they are widely used in the prevention and treatment of intestinal diseases in various animals (Lan et al., 2017; Jazi et al., 2018; Mârza et al., 2025). Currently, there is limited research on the effects of their combined use, and further validation is necessary. In addition, previous studies have indicated that both strains allow relatively efficient nanoparticle internalization compared with other lactic acid bacteria, providing a practical advantage for future delivery-based applications (Kim et al., 2018; Mohammed et al., 2021). Taken together, these complementary features and shared suitability for nanotechnology provide a rationale for selecting these two strains over other commonly used probiotics.

In the present study, *L. acidophilus*, *P. acidilactici* and their different ratios of those two lactic acid bacteria were given to mice with colitis. Therefore, this study aimed to investigate the protective effects of a combined administration of *L. acidophilus* and *P. acidilactici* at different ratios in a DSS-induced colitis mouse model.

## 2 Materials and methods

### 2.1 Bacterial strains

*L. acidophilus* (BNCC 186473) and *P. acidilactici* (BNCC 137601) were obtained as frozen vials from BNCC (Beijing, China). The strains were routinely cultured in MRS broth at 37 °C under static conditions. They were maintained by revival and subculturing according to the BNCC frozen-culture protocol and stored at −80 °C in MRS supplemented with 15% (w/v) glycerol.

For *in vivo* administration, both strains were cultured in MRS broth at 37 °C and harvested for 10 h prior to centrifugation (3,000 × g for 3 min at 4 °C), washed twice with sterile phosphate buffered saline (PBS), and resuspended in PBS to reconstituted to a density of approximately 5 × 10^8^ CFU/mL, as ascertained through colony counting on MRS plate, in preparation for subsequent experiments. Mouse received an oral administration of 1 × 10^8^ CFU in 0.2 mL via oral gavage daily.

### 2.2 Experimental animal

All animal procedures were performed in compliance with the Guidelines for the Ethics and Use of Agricultural Animals in Research and Teaching, as approved by the experimental animal ethics committee of Yanbian University (ethical review acceptance number: YD20220718003).

C57BL/6 male mice (7–8 weeks old) were purchased from the experimental animal center of Yanbian University. The mice were housed under specific pathogen-free conditions with controlled temperature (23 ± 2 °C), humidity (55–65%), and a 12 h light/dark cycle. The mice were provided with *ad libitum* access to a standard chow diet and water.

**Figure 1:**
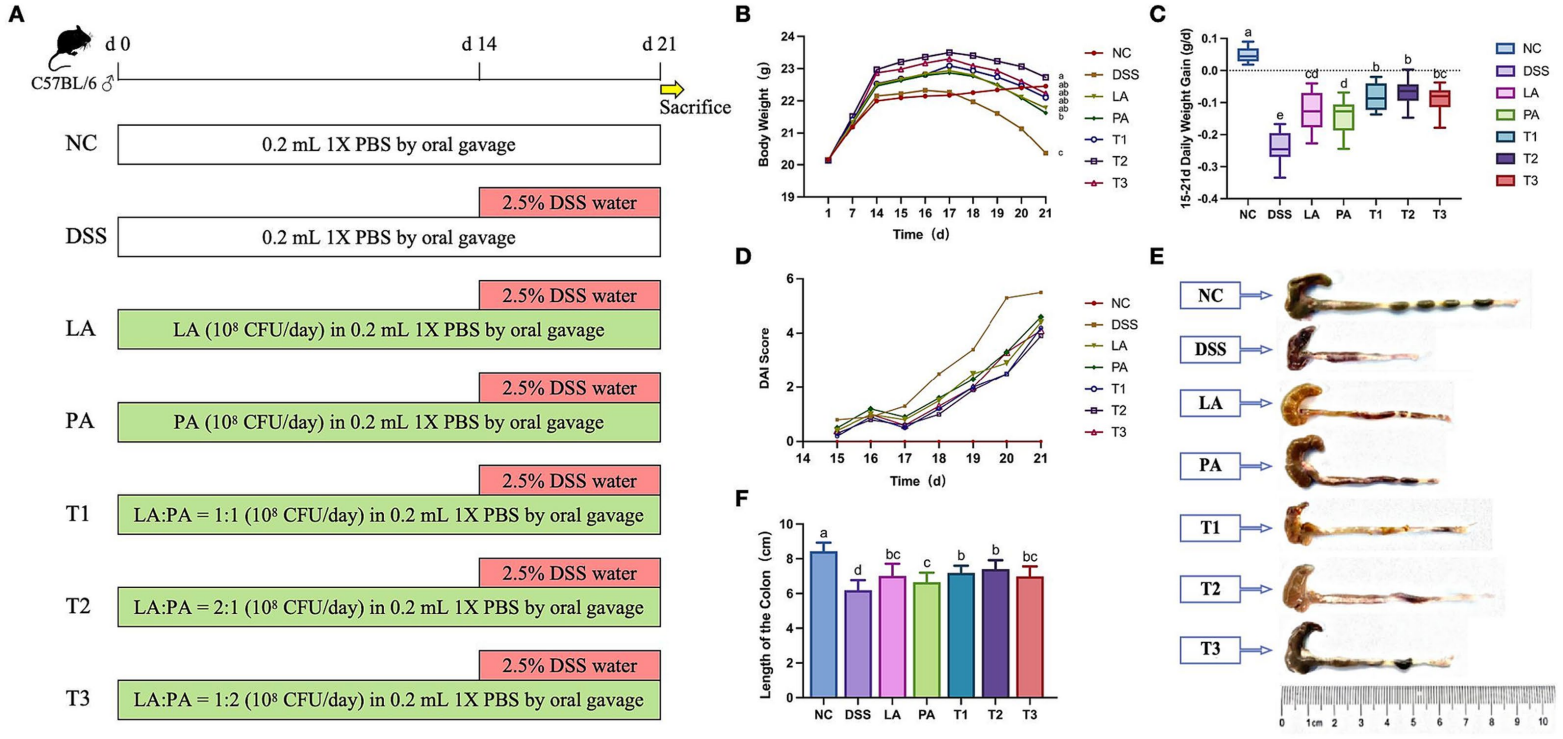
The alleviating effect of *L. acidophilus* combined with *P. acidilactici* on body weight, disease activity index (DAI) score, colon morphology, and colon length. A group of mice were treated with LA, PA, T1, T2, and T3 from day 1 to 21, with colitis induced by 2.5% DSS in drinking water from day 15 to 21. Clinical symptoms were monitored daily, and colonic tissues were harvested immediately after sacrifice. **(A)** Schematic diagram showing experimental design. **(B)** Weight changes in mice from day 1 to 21. **(C)** Changes in daily weight gain. **(D)** DAI in mice from day 15 to 21. **(E,F)** Dextran sodium sulfate (DSS) was administered for 7 days, and **(E)** colon morphology and **(F)** colon length were recorded. Data are shown as the mean value ± standard error of the mean (*n* = 10). Different letters in each panel indicate significant differences at *p* < 0.05. LA, *Lactobacillus acidophilus*; PA, *Pediococcus acidilactici*; T1, *Lactobacillus acidophilus: Pediococcus acidilactici* = 1: 1; T2, *Lactobacillus acidophilus: Pediococcus acidilactici* = 2: 1; T3, *Lactobacillus acidophilus: Pediococcus acidilactici* = 1: 2.

During the 7-day adaptation period, all mice were allowed to acclimatize without any treatment. After the adaptation period, the experiment was divided into a 14-day feeding period and a 7-day colitis induction period (Figure 1A). Seventy mice were randomly divided into seven groups (*n* = 10 per group): negative control group (NC), positive control group (DSS, colitis induced by dextran sulfate sodium (DSS) treatment), *L. acidophilus* group (LA), *P. acidilactici* group (PA), *L. acidophilus*: *P. acidilactici* = 1: 1 group (T1), *L. acidophilus: P. acidilactici* = 2: 1 group (T2), and *L. acidophilus: P. acidilactici* group = 1: 2 (T3). During the feeding period (days 1–14), the treatment groups (including single and combined probiotic groups) were given an oral gavage of 1 × 10^8^ CFU of *L. acidophilus*, *P. acidilactici*, or their combination (T1, T2, and T3 ratios as defined above) in 200 μL PBS daily. Then, colitis was induced by administration of drinking water containing 2.5% DSS (MW 36,000–50,000 Da) for 7 consecutive days (days 15–21), while the probiotic administration continued in the same way as during the feeding period.

### 2.3 Blood and tissue collection

At the end of the experiment, the mice were anesthetized by inhalation of 3% isoflurane and the blood samples were collected by orbital puncture using a capillary tube and left to stand at low temperature for 2 h until stratification. The samples were then centrifuged at 3,500 × g for 10 min at 4 °C to separate the serum, which was stored at −20 °C until further analysis. Then, the mice were euthanized by CO_2_ inhalation with a displacement rate of 20% chamber volume/min and the colon samples were collected and its length measured.

### 2.4 Disease activity index

The disease activity index (DAI) of mice is evaluated by three aspects, which reflects the severity of colitis. Changes in body weight, fecal traits and hematochezia of the mice were recorded every day during the experiment. DAI was calculated by summing the scores for weight loss, stool consistency, and the degree of hematochezia for each mouse (Cooper et al., 1993).

### 2.5 Histology

Following sample collection, 1 cm of the middle colon tissue was dipped in a 4% paraformaldehyde solution and fixed for 24 h. Then, the colon tissue was excised for trimming, and the mesentery along with other attachments were removed. Dehydration, routine paraffin embedding, and H&E staining were conducted.

Intestinal morphology was evaluated by measuring mucosal thickness, crypt depth, and the ratio of mucosal thickness to crypt depth. Mucosal thickness is defined as the vertical distance from the luminal epithelial surface to the muscularis mucosae. Crypt depth is defined as the vertical distance from the crypt opening to its base.

### 2.6 Antioxidant indexes

The kits (Nanjing Institute of Bioengineering, Nanjing, China) were used to determine the amounts of malonaldehyde (MDA), myeloperoxidase (MPO), nitric oxide synthase (NOS), superoxide dismutase (SOD), glutathione peroxidase (GSH-Px), and total antioxidant capacity (T-AOC).

### 2.7 mRNA expression of tight junction proteins in colonic mucosa

Colon tissue was homogenized in a centrifuge tube, and total RNA was extracted from the colon using the Trizol method. cDNA synthesis was performed using a cDNA reverse transcription kit (Shanghai Enzyme Biotechnology Co., Ltd., China). The three-step real-time PCR reaction program was as follows: 5 μL SYBR Green, 0.8 μL primers (10 μmol/L), 1 μL cDNA (1,000 ng/μL), and distilled water to make up a 10 μL system. The reaction conditions included preheating at 95 °C for 30 s, followed by the cycling phase consisting of denaturation at 95 °C for 5 s, annealing at 59 °C for 1 min, and extension at 72 °C for 30 s, repeated for 40 cycles. The melting curve was conducted at 65 °C for 5 s, followed by 95 °C for 5 s. The 2(−ΔΔCt) method, using Gapdh as the internal control, was used to quantify the mRNA levels of inflammation-related genes in colon tissue. The sequences of target gene primers are shown in Table 1.

**Table 1:** Sequence of gene primer set.

Target gene	Primer sequence (5′-3′)
*Gapdh*	F: TGCACCACCAACTGCTTAG
	R: GGATGCAGGGATGATGTTC
*ZO-1*	F: CCTGTGAAGCGTCACTGTGT
	R: CGCGGAGAGAGACAAGATGT
*Occludin*	F: CATAGTCAGATGGGGGTGGA
	R: ATTTATGATGAACAGCCCCC

### 2.8 ELISA assays to measure cytokines

Levels of interleukin (IL)-1β, IL-6, and tumor necrosis factor (TNF)-α in colonic tissue homogenates were measured using ELISA kits (all from Jiangsu Enzyme-Labeled BioTECH, Yancheng, China) according to the manufacturer’s instructions.

### 2.9 Gut microbiota analysis

Genomic DNA was extracted from samples using the cetyltrimethylammonium bromide (CTAB) method. The V4 region of the bacterial 16S rRNA gene was amplified using specific primers 515F (5′-GTGCCAGCMGCCGCGGTAA-3′) and 806R (5′-GGACTAC HVGGGTWTCTAAT-3′). The PCR amplification was performed under the following conditions: initial denaturation at 98 °C for 1 min; followed by 30 cycles of denaturation at 98 °C for 10 s, annealing at 50 °C for 30 s, and extension at 72 °C for 30 s, with a final extension at 72 °C for 5 min. The PCR products were purified using a Qiagen gel extraction kit (QIAGEN, Germany) and quantified by fluorescence-based techniques. The purified DNA was used to construct sequencing libraries with the TruSeq® DNA PCR-free sample preparation kit (Illumina, United States). The library quality was assessed using a Qubit® 2.0 Fluorometer (Thermo Scientific) and an Agilent Bioanalyzer 2100 system. Paired-end sequencing (2 × 250 bp) was performed using the Illumina NovaSeq platform (Novogene, China). Raw sequencing data were processed with the QIIME software package (v1.9.1).

### 2.10 Determination of SCFAs

The concentration of SCFAs was measured by the external standard method using a gas chromatograph (GC-7890A, Agilent Technologies Inc., Santa Clara, CA, United States). The injection volume was set at 1 μL, with injector and detector temperatures set at 170 °C and 200 °C, respectively. The column temperature was maintained at 120 °C and nitrogen was used as the carrier gas at a flow rate of 26.43 mL/min. For sample preparation, the fermentation supernatant was centrifuged at 13,400 × g for 10 min. Then, 0.8 mL of the supernatant was transferred into a 1.5 mL centrifuge tube containing 0.2 mL of 25% metaphosphoric acid. Then, the mixture was allowed to stand for 30 min, followed by centrifugation at 10,000 × g for 15 min. The resulting supernatant was filtered through a 0.22 μm membrane before GC analysis. Quantification was performed using the external standard method with five-point calibration curves constructed for each SCFA. The linearity of the calibration curves was robust, with all correlation coefficients at R^2^ > 0.99.

### 2.11 Statistical analyses

The data obtained in the above experiments were statistically analyzed by SPSS 17.0 software. One-way ANOVA for analysis of variance, and LSD and Duncan methods were used for pairwise comparison, *p* < 0.05 was considered as a significant level, and data were expressed as mean ± standard deviation. Bar plots were generated by using GraphPad Prism 10.2.3. To integrate related host biomarkers into a comprehensive analysis, principal component analysis (PCA) was performed. Prior to PCA, all variables were Z-score standardized to eliminate scaling differences. PCA was conducted on the protein concentrations of TNF-α, IL-1β, and IL-6 from colonic tissue homogenates and separately on the data from six antioxidant markers (SOD, GSH-Px, T-AOC, MPO, MDA, and NOS). The first principal component (PC1), was extracted to serve as a variable for subsequent correlation analysis. To explore the intrinsic associations among the microbiota, metabolites, and host phenotypes, a Spearman’s rank correlation analysis was performed based on all individual sample data. The variables analyzed included the relative abundance of *Akkermansia*, cecal butyrate concentration, the PC1 scores derived from cytokines and antioxidant markers, and the relative mRNA expression of *ZO-1* and *occludin*. The correlation matrix (Spearman r) and corresponding *p*-values are presented as a heatmap and scatter plots of key significant correlations in the supplementary materials. Key significant correlations were highlighted and plotted as scatter plots. Significance levels are denoted as follows: *p < 0.05, **p < 0.01, and ***p < 0.001.

## 3 Results

### 3.1 Combination of *Lactobacillus acidophilus* and *Pediococcus acidilactici* improves body weight changes in mice with colitis

Weight loss is a well-established and objective parameter of DSS-induced colitis in mice (Britto et al., 2019). To evaluate whether the combined probiotics ameliorate colitis symptoms, we monitored body weight (Figure 1B) and daily weight gain (Figure 1C). From days 15 to 21 of the experiment, the daily weight gain in all DSS-treated groups decreased compared to the NC group due to the progression of colitis. At day 21, the body weight of the T2 group was significantly higher than that of the DSS group (*p* < 0.05); all other groups significantly alleviated the decrease in daily weight gain (*p* < 0.05). Overall, the T2 group showed less weight loss compared to the other groups.

### 3.2 Combination of *Lactobacillus acidophilus* and *Pediococcus acidilactici* alleviates DAI score and restores colonic tissue integrity in mice with colitis

To further assess inflammation severity and tissue damage, we evaluated DAI scores, colon length, and histological changes. The DAI scores (Table 2) showed the severity of colitis, including the degree of weight loss, the presence of blood in the stool, and stool consistency. The DSS group mice showed a significant increase in the index of DAI score on the second day of the experiment (Figure 1D). Compared to the DSS group, the DAI indexes of the mice in each treatment group showed a reduction. Severe inflammation may result in symptoms such as bloody stool, leading to completely unformed colon contents. The colon contents, while not distinctly granular, remained well-formed, and the presence of bloody stool showed significant improvement in the T2 group (Figure 1E).

**Table 2:** DAI scoring criteria.

Score	Weight loss	Stool consistency	Degree of hematochezia
0	<1%	Normal	Normal
1	1–5%	Slightly loose but tangible	Mild occult blood
2	6–10%	Severely loose and semi-formed	Occult blood
3	11–15%	Loose stool	Hematochezia
4	>15%	Heavier loose stool	Severe hematochezia

As expected, there was a significant (*p* < 0.05) reduction in colon length in the DSS group. All treatment groups significantly alleviated DSS-induced colon shortening in mice compared to the DSS group (*p* < 0.05). The colon length in the T2 group was significantly greater than in the DSS group (*p* < 0.05) and showed the most substantial improvements compared to other treatment groups, although it was not statistically different from the other combination groups (Figures 1E,F). The T2 group demonstrated superior efficacy compared to other treatment groups in alleviating DSS-induced intestinal injury.

**Figure 2:**
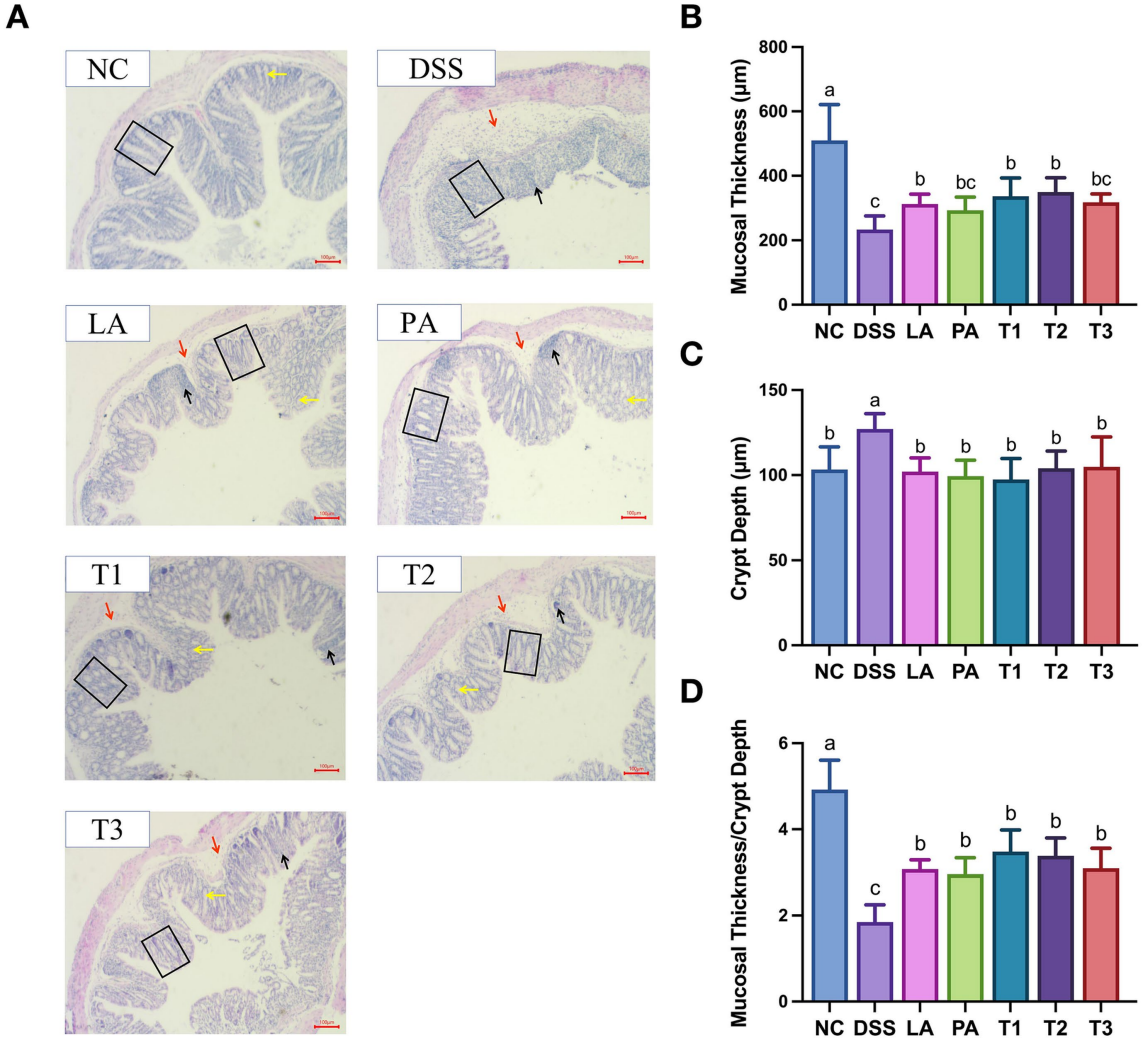
The alleviating effect of *L. acidophilus* combined with *P. acidilactici* on colonic tissue injury. Middle colon tissue was fixed in 4% paraformaldehyde, embedded in paraffin, and sectioned for H&E staining. Histological structures were examined under light microscopy **(A)**. Representative images of H&E-stained colonic sections (10-fold magnification; scale bar = 100 μm) **(B)**. Mucosal thickness **(C)**. Crypt depth **(D)**. Mucosal thickness/crypt depth. Data are shown as the mean value ± standard error of the mean (*n* ≥ 5). Different letters in each panel indicate significant differences at *p* < 0.05. Black arrows indicate inflammatory cell infiltration. Red arrows denote submucosal edema. Yellow arrows indicate goblet cells. Black boxes outline the morphology of colonic crypts. DSS, dextran sodium sulfate; LA, *Lactobacillus acidophilus*; PA, *Pediococcus acidilactici*; T1, *L. acidophilus: P. acidilactici* = 1: 1; T2, *L. acidophilus: P. acidilactici* = 2: 1; T3, *L. acidophilus: P. acidilactici* = 1: 2.

Next, we examined the effect of *L. acidophilus* combined with *P. acidilactici* on colonic pathology in mice (Figure 2). As expected, Figure 2A showed that the NC group had well-developed mucosa, normal crypts, intact gland structures, and abundant goblet cells with no evidence of immune cell infiltration. The DSS group exhibited significant mucosal epithelium damage, characterized by extensive gland destruction, loss of crypt structures, submucosal edema, and substantial immune cell infiltration. It was noting that the inflammatory condition improved across all treatment groups receiving lactic acid bacteria. The T2 group showed the most significant improvement in the alleviation of pathological structural damage to the colon. The vast majority of epithelial structures retained their integrity, with a reduction in the destruction of crypts and glandular structures, along with a significant decrease in the cell infiltration after 7 days of DSS induction.

Figures 2B–D illustrates the effects of *L. acidophilus* combined with *P. acidilactici* on the thickness of colon mucosa and the depth of the crypts, and their respective ratio. The DSS group had a significantly greater crypt depth than the NC group (*p* < 0.05). After intervention with lactic acid bacteria, all treatment groups showed effective relief, with no significant difference observed compared to the NC group (*p* > 0.05). The mucosal thickness and the ratio of mucosal thickness to crypt depth in the DSS group were significantly lower than those in the NC group (*p* < 0.05). Following intervention with lactic acid bacteria, all treatment groups showed improvement, with the T1 and T2 groups performing better improvement in mucosal thickness compared to other treatment groups.

### 3.3 Combination of *Lactobacillus acidophilus* and *Pediococcus acidilactici* attenuates serum oxidative stress in mice with colitis

**Figure 3:**
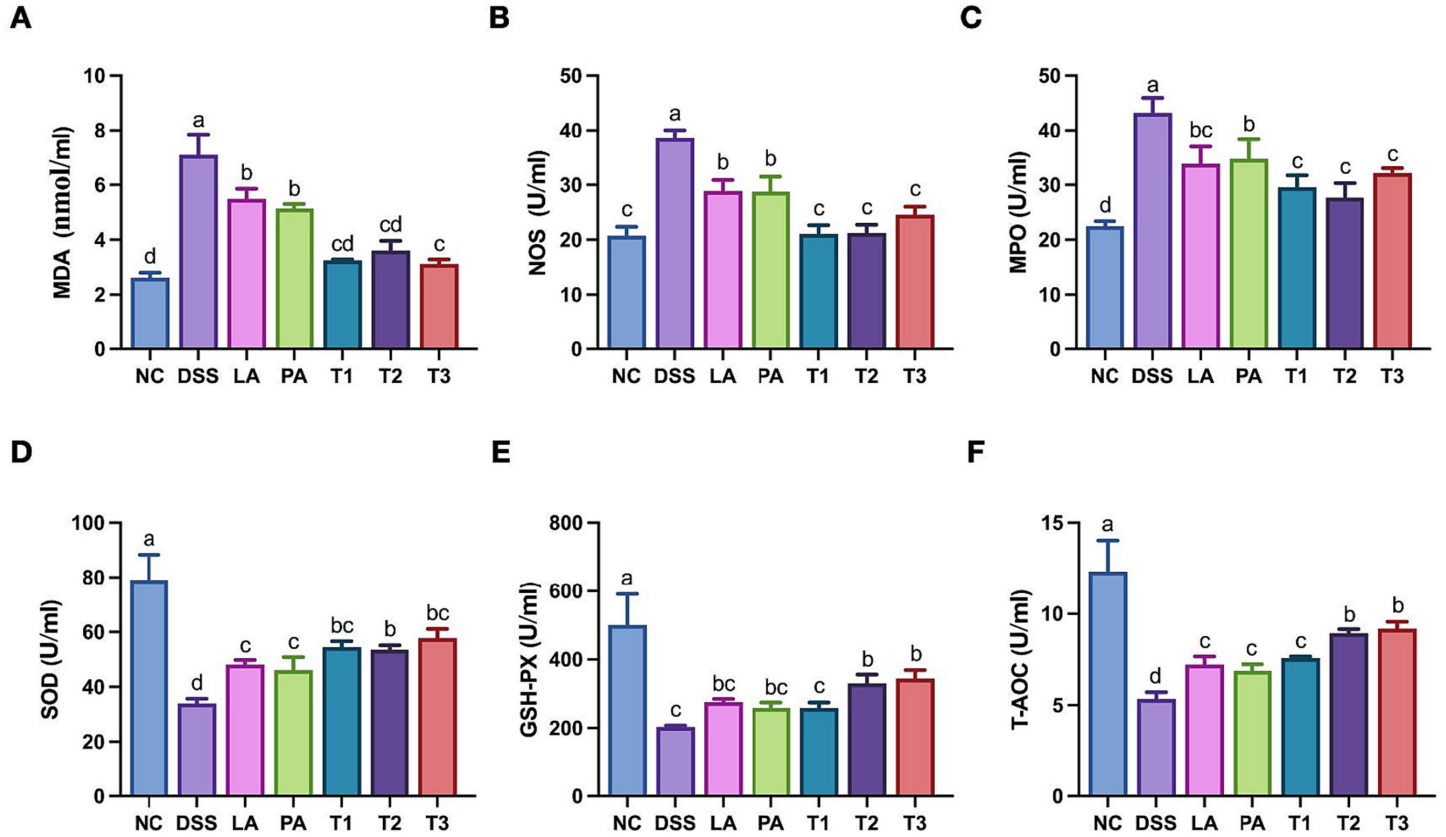
Serum antioxidant indexes in colitis-induced mice treated with *L. acidophilus* and *P. acidilactici*. Blood samples were collected to obtain serum. Oxidative stress markers and antioxidant enzyme activities were determined using commercial assay kits. The content of serum **(A)** MDA; **(B)** NOS; **(C)** MPO; **(D)** SOD; **(E)** GSH-PX; **(F)** T-AOC from mice with IBD. Data are shown as the mean value ± standard error of the mean (*n* ≥ 5). Different letters in each panel indicate significant differences at *p* < 0.05. DSS, dextran sodium sulfate; LA, *L. acidophilus*; PA, *P. acidilactici*; T1, *L. acidophilus: P. acidilactici* = 1: 1; T2, *L. acidophilus: P. acidilactici* = 2: 1; T3, *L. acidophilus: P. acidilactici* = 1: 2.

Oxidative stress is a major contributor to intestinal tissue injury and has been widely implicated in the pathogenesis of inflammatory bowel disease (Jin et al., 2025). Given that local intestinal inflammation often leads to systemic redox imbalance, we investigated the systemic antioxidative effects of the combined probiotics. Key oxidative markers in the serum were quantified to evaluate the host’s overall redox status. The effects of *L. acidophilus* combined with *P. acidilactici* on serum oxidative stress in mice with DSS-induced colitis are shown in Figure 3. The induction of DSS significantly decreased the SOD, T-AOC, and GSH-PX levels in the mice (*p* < 0.05). The intervention with combined lactic acid bacteria increased all indicators, with the levels of serum SOD, T-AOC, and GSH-PX in groups T2 and T3 significantly higher than those in the DSS group (*p* < 0.05). Following treatment with combined lactic acid bacteria, significant improvements were observed, particularly in group T2, which exhibited the most favorable outcomes, as all indicators showed significant reduction compared to the DSS group.

### 3.4 Combination of *Lactobacillus acidophilus* and *Pediococcus acidilactici* suppresses the expression of inflammatory cytokines in DSS-induced colitis mice

**Figure 4:**
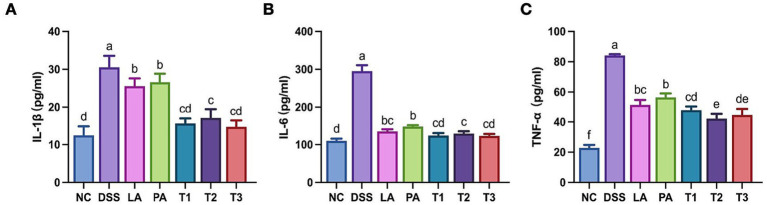
Inflammatory indexes of colonic mucosa in colitis-induced mice treated with *L. acidophilus* and *P. acidilactici*. Colonic tissues were homogenized in cold PBS, and supernatant cytokine concentrations were measured using enzyme-linked immunosorbent assay (ELISA) kits. Protein expression levels of **(A)** IL-1β, **(B)** IL-6, and **(C)** TNF-α in colonic mucosa are displayed. Data are shown as the mean value ± standard error of the mean (*n* ≥ 5). Different letters in each panel indicate significant differences at *p* < 0.05. DSS, dextran sodium sulfate; LA, *Lactobacillus acidophilus*; PA, *Pediococcus acidilactici*; T1, *L. acidophilus: P. acidilactici* = 1: 1; T2, *L. acidophilus: P. acidilactici* = 2: 1; T3, *L. acidophilus: P. acidilactici* = 1: 2.

Next, we examined the impact of the combined probiotics on the inflammatory response associated with colitis by measuring pro-inflammatory cytokine levels in colonic tissues. Elevated pro-inflammatory cytokines are indicative of inflammation and are correlated with the severity of colitis (Zhang et al., 2022). Figures 4A–C illustrates the impact of *L. acidophilus* and *P. acidilactici* on inflammatory cytokines in mice with DSS-induced colitis. In comparison to the NC group, the levels of inflammatory cytokines in the DSS group significantly elevated (*p* < 0.05). Following intervention with lactic acid bacteria, all treatment groups exhibited a significant (*p* < 0.05) reduction in IL-1β, IL-6, and TNF-α at the protein level, with the combined groups demonstrating the most pronounced effect.

### 3.5 Combination of *Lactobacillus acidophilus* and *Pediococcus acidilactici* upregulates mRNA expression of intestinal tight junction proteins in mice with colitis

**Figure 5:**
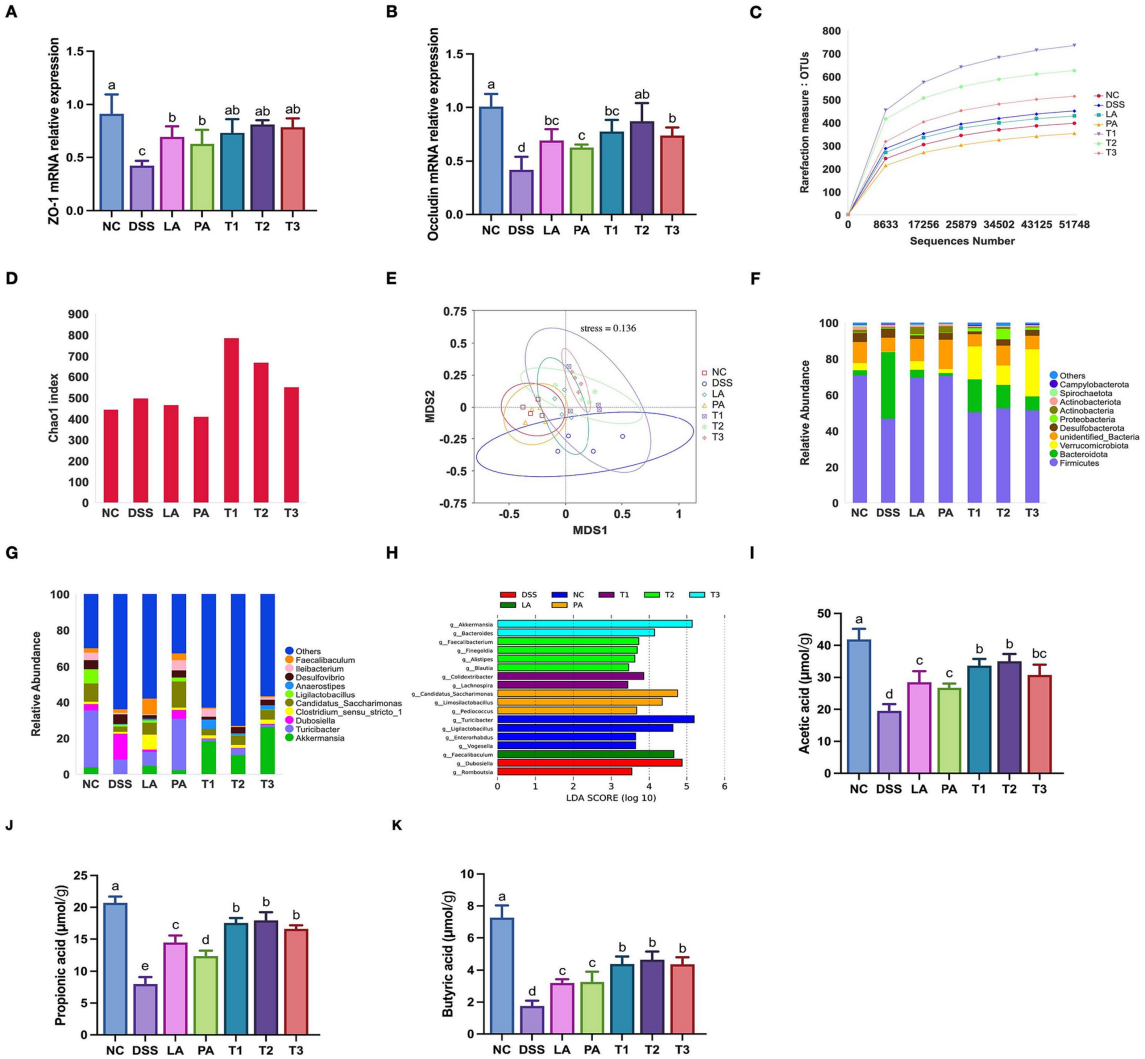
The treatment of *L. acidophilus* combined with *P. acidilactici* on improves tight junction proteins, gut microbiota, and cecal SCFAs in DSS-induced colitis mice. Colonic tight junction markers were assessed via RT-qPCR. Cecal contents were collected for 16S rRNA gene sequencing to analyze microbial community structure, and SCFAs were quantified using gas chromatography. **(A)** mRNA expression of zonula occludens-1 (*ZO-1*) in mice colonic mucosa; **(B)** mRNA expression of *occludin* in mice colonic mucosa; **(C)** Rarefaction curve; **(D)** α-diversity estimated by the Chao 1 index; **(E)** NMDS plot based on the weighted_unifrac index was used to assess β-diversity; Relative abundances of the gut microbial community at the phylum level **(F)** and the genus level **(G)**; **(H)** Linear discriminant analysis (LDA) effect size (LEfSe) analysis (threshold > 3.5); **(I)** Acetic acid in cecal contents; **(J)** Propionic acid in cecal contents; **(K)** Butyric acid in cecal contents. Data are shown as the mean value ± standard error of the mean (*n* ≥ 4). Different letters in each panel indicate significant differences at *p* < 0.05. DSS, dextran sodium sulfate; LA, *Lactobacillus acidophilus*; PA, *Pediococcus acidilactici*; T1, *L. acidophilus: P. acidilactici* = 1: 1; T2, *L. acidophilus: P. acidilactici* = 2: 1; T3, *L. acidophilus: P. acidilactici* = 1: 2.

Tight junction proteins are essential for maintaining intestinal epithelial barrier integrity, disruption of these proteins is considered a critical feature of colitis (Chelakkot et al., 2018b). We examined the expression of tight junction proteins to evaluate the effect of the treatment on mucosal barrier integrity. Figures 5A,B illustrates the impact of *L. acidophilus* and *P. acidilactici* on the mRNA expression of colonic tight junction proteins in mice with DSS-induced colitis. In comparison to the NC group, the DSS group exhibited a significant reduction in the relative mRNA expression levels of *ZO-1* and *occludin* (*p* < 0.05). Following intervention with lactic acid bacteria, all treatment groups exhibited significant increases in gene expression (*p* < 0.05), with the T2 group demonstrating the most pronounced effect.

### 3.6 Combination of *Lactobacillus acidophilus* and *Pediococcus acidilactici* modulates gut microbiota and metabolites in mice with DSS-induced colitis

Next, we further explored the underlying regulatory mechanisms by analyzing changes in the gut microbiota and metabolites. Gut microbiota dysbiosis and changes of SCFA production play a critical role in the development of colitis by disrupting barrier integrity and promoting inflammation (Haneishi et al., 2023). Figure 5C illustrates that the rarefaction curves for all samples reached a plateau, indicating that the current sequencing depth was sufficient to encompass microbial diversity, thus confirming the reliability of the sequencing data. Analyses of alpha and beta diversity are presented in Figures 5D,E. Compared to the NC group, the DSS group showed an increasing trend in the Chao1 index. Following intervention with lactic acid bacteria, the Chao1 indexes of the T1, T2, and T3 groups exhibited a substantial elevation (*p* < 0.05) compared to the DSS group, with T2 showing the most pronounced increase. The Chao1 indices for the LA and PA groups, however, exhibited a decline (*p* > 0.05). The NMDS results demonstrated that DSS caused substantial changes in gut microbial composition. The microbial communities in colitis-induced mice treated with combined lactic acid bacteria were distinctly clustered in contrast to mice with colitis.

The impact of *L. acidophilus* combined with *P. acidilactici* on relative abundance is shown in Figures 5F,G, where the relative abundance of gut microbiota at both the phylum and genus levels was analyzed. The predominant phyla identified were Firmicutes, Bacteroidota, and Verrucomicrobiota. Compared to the NC group, the DSS group showed a significant (*p* < 0.05) reduction in the relative abundance of Firmicutes and an increase in Bacteroidota. Following the intervention with lactic acid bacteria, these trends were reversed, resulting in an increase in Firmicutes and an a decrease in Bacteroidota. In addition, colitis induction significantly diminished Verrucomicrobiota (*p* < 0.05); however, its relative abundance was increased in all treatment groups upon intervention with lactic acid bacteria. The T1, T2, and T3 groups exhibited significantly elevated levels of Verrucomicrobiota when compared to the DSS group (*p* < 0.05). After intervention with lactic acid bacteria, the prevalence of *Akkermansia* increased in all treatment groups, with the T1, T2, and T3 groups showing significantly higher levels than the DSS group (*p* < 0.05).

Figure 5H shows that LEfSe analysis identified significant differences in biomarkers at the genus level among several treatment groups (LDA score > 3.5, *p* < 0.05). The NC group exhibited a high prevalence of *Turicibacter*, *Ligilactobacillu*s, and *Enterorhabdus*. The DSS group had an abundance of *Dubosiella* and *Romboutsia*. The LA group showed enrichment of *Faecalibaculum*, while the PA group was enriched with *Candidatus_Saccharimonas* and *Limosilactobacillus*. Among the combined lactic acid bacteria intervention groups, the T1 group was significantly enriched in *Akkermansia* and *Bacteroide*s; the T2 group in *Colidextribacter* and *Lachnospira*; and the T3 group in *Faecalibacterium*, *Alistipes*, and *Blautia*.

As shown in Figures 5I–K, the cecal concentrations of acetic acid, propionic acid and butyric acid were significantly decreased in the DSS group compared to the NC group (*p* < 0.05). Following the intervention in mice with lactic acid bacteria, all treatment groups showed significantly increased butyric acid levels (*p* < 0.05), with the T2 group demonstrating the most substantial improvement.

### 3.7 Correlation analysis reveals strong associations among microbiota, metabolites, and host phenotypes

**Figure 6:**
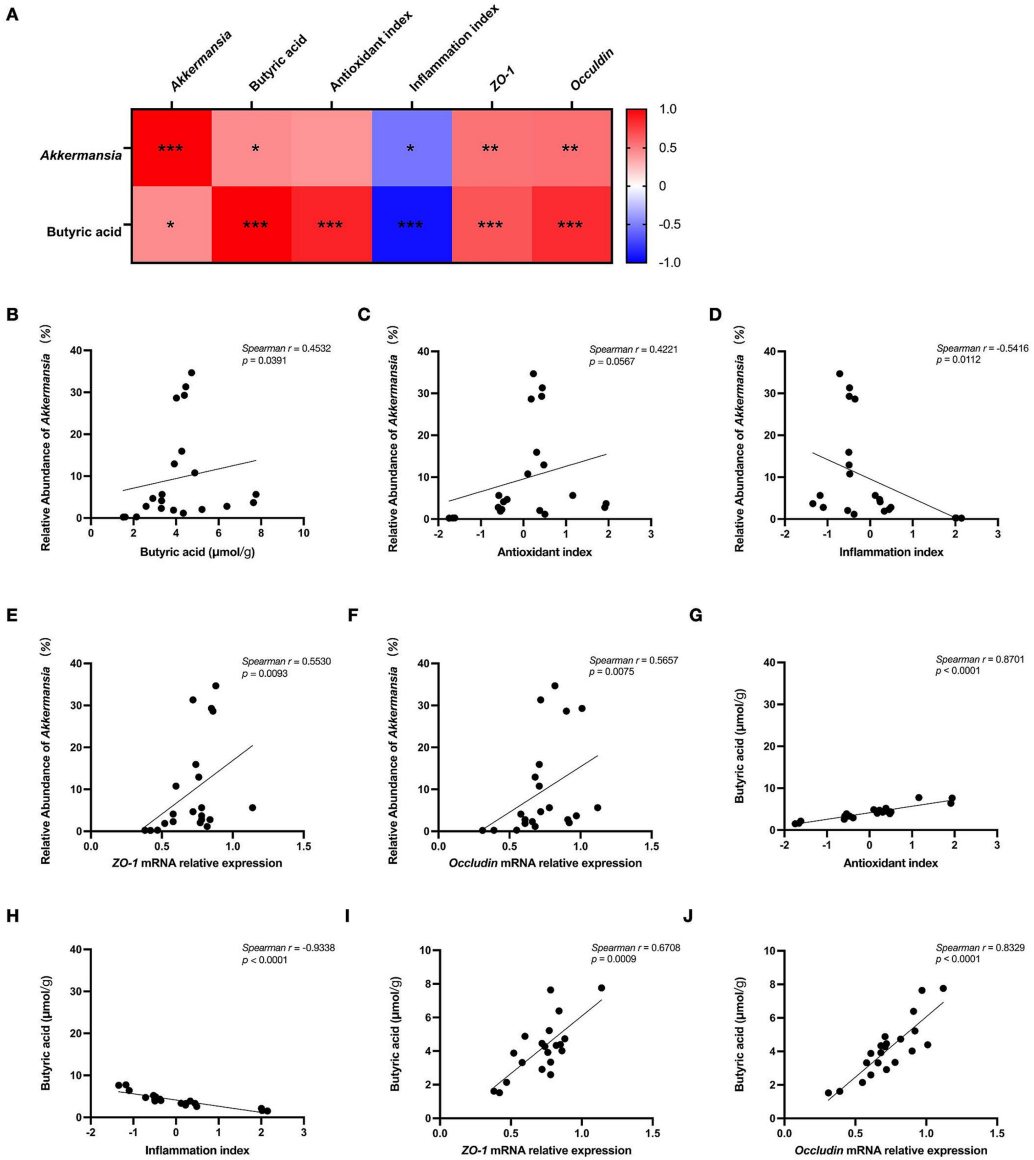
Correlation analysis among microbial, metabolite, and host physiological indexes. Data obtained from microbial sequencing, SCFA quantification, and host physiological parameters were integrated, and pairwise associations were evaluated using Spearman’s rank correlation analysis. Spearman’s rank correlation analysis was performed. **(A)** Spearman correlation heatmap between *Akkermansia*, butyric acid and *Akkermansia*, butyric acid, antioxidant index, inflammation index, and tight junction proteins gene expression in all groups. Positive correlations are shown in red, and negative correlations in blue. Scatter plots of key significant correlations. **(B)** *Akkermansia* relative abundance vs. butyrate concentration; **(C)** *Akkermansia* relative abundance vs. antioxidant index (PC1); **(D)** *Akkermansia* relative abundance vs. inflammation index (PC1); **(E)** *Akkermansia* relative abundance vs. *ZO-1* expression; **(F)** *Akkermansia* relative abundance vs. *occludin* relative expression; **(G)** Butyrate concentration vs. antioxidant index (PC1); **(H)** Butyrate concentration vs. inflammation index (PC1); **(I)** Butyrate concentration vs. *ZO-1* relative expression; **(J)** Butyrate concentration vs. *occludin* relative expression. Spearman’s *r* and *p*-values are indicated on each plot (*n* ≥ 3). Numerical values within cells represent the *ρ* coefficient. Significance levels are indicated as follows: *p < 0.05, **p < 0.01, ***p < 0.001.

Given that the combined probiotic treatments enriched the relative abundance of *Akkermansia* and elevated cecal butyrate levels, we sought to investigate whether these specific microbiota and metabolite changes were statistically linked to the improvements in host phenotypes. A Spearman’s rank correlation analysis was performed on all individual animal samples in Figure 6. Principal component analysis (PCA) was performed to integrate host phenotypes. The first principal component (PC1) extracted from cytokines explained 86.943% of the total variance and exhibited strong positive loadings for all three pro-inflammatory cytokines, indicating that higher concentrations of these cytokines contribute to a higher PC1 score. Therefore, the PC1 score was defined as the “Inflammation Index,” where a higher score represents a stronger overall inflammatory status. Similarly, for antioxidant markers, the PC1 extracted explained 84.729% of the total variance. This PC1 score correlated positively with antioxidant enzymes (SOD, GSH-Px, T-AOC) and negatively with oxidative products (MPO, MDA, NOS). Thus, the PC1 score was defined as the “Antioxidant Index,” where a higher score represents a stronger overall antioxidant capacity. The overall correlation heatmap revealed a distinct pattern of interactions (Figure 6A). As shown in Figures 6B–F illustrates that, at the microbial level, the relative abundance of *Akkermansia* is significantly positively correlated with cecal butyrate concentration (*p* < 0.05). This finding suggests a potential synergistic relationship in which the colonization of *Akkermansia* may favor a butyrogenic environment. The abundance of *Akkermansia* was also significantly negatively correlated with the inflammation index (*p* < 0.05), indicating that the restoration of *Akkermansia* is closely tied to the alleviation of inflammatory signaling. It also reached statistical significance for direct correlation with both tight junction genes, *ZO-1* and *occludin* (*p* < 0.01), implying a potential role of *Akkermansia* in reinforcing the mucosal physical barrier.

Parallel to the microbiota findings, cecal butyrate concentration has been identified as a key metabolite regulator, showing robust correlations with host phenotypes (Figures 6G–J). As illustrated in the scatter plots, there was a significant positive correlation between butyrate and the antioxidant index (*p* < 0.001) and an apparent negative correlation with the inflammation index (*p* < 0.001). Furthermore, increased butyrate levels were positively correlated with the relative mRNA expression of both tight junction protein genes, *ZO-1* (*p* < 0.001) and *occludin* (*p* < 0.001). The strong associations indicate that butyrate serves as a central metabolite hub for host-protective effects, linking probiotic-induced modulation of the microbiota to the observed enhancement in gut integrity, antioxidant capacity, and immune homeostasis.

## 4 Discussion

Probiotics might offer therapeutic potential for colitis; however, the efficacy of individual strains is often variable due to colonization resistance or limited metabolic functions (Liu et al., 2025). We selected *L. acidophilus* and *P. acidilactici* to address potential niche complementarity. *L. acidophilus* shows significant epithelial adhesion (Goh and Klaenhammer, 2010), whereas *P. acidilactici* produces bacteriocin that helps modify the gut environment (Parada et al., 2007). This combination theoretically establishes a synergistic protective mechanism in which *L. acidophilus* physically protects mucosal niches while *P. acidilactici* chemically modulates the luminal environment, enhancing overall colonization stability. This dual-action model theoretically creates a protective synergy that exceeds the capacity of individual strains. Consistent with other reports, our findings demonstrated that the combination of certain microbiota markedly alleviated DSS-induced colitis and displayed a stronger protective effect than either strain alone (Hua et al., 2025). The improvements were evidenced by the preservation of histological architecture and reduced disease activity. Such synergistic efficacy appears to be driven by reshaping the gut ecosystem, specifically pointing to a potential cross-feeding loop between *Akkermansia* and butyrate. Therefore, this specific probiotic combination represents a promising candidate for colitis management.

Excessive activation of the NF-κB pathway is central to IBD pathogenesis, promoting induction of key pro-inflammatory cytokines, such as TNF-α, IL-6, and IL-1β (Mukherjee et al., 2024). In the present study, we have demonstrated that the combined *L. acidophilus* and *P. acidilactici* intervention significantly reduced the cytokine surge, demonstrating superior efficacy compared to each strain. This aligns with the reduced inflammatory cell infiltration observed in colonic tissues, thus confirming a significant anti-inflammatory efficacy. Recent findings in weaning piglets indicate that multi-strain probiotic supplementation has the potential to modulate the immune balance by upregulating anti-inflammatory cytokines, such as IL-4 and IL-10 (Sahatsanon et al., 2024).

Chronic intestinal inflammation concurrently generates excessive reactive oxygen species (ROS), causing oxidative stress that disrupts epithelial integrity and amplifies inflammatory responses (Zhao et al., 2023; Muro et al., 2024). Barrier disruption facilitates the entry of luminal pro-oxidants into the circulation, intensifying systemic oxidative stress (Odenwald et al., 2016). Therefore, we have examined serum oxidative markers to assess systemic redox status. The results indicated that DSS challenge induced severe oxidative damage, evidenced by elevated MDA, NOS, and MPO levels and reduced SOD, GSH-Px, and T-AOC activities as expected (Zhang et al., 2023). Our results demonstrated that the combinatory probiotic intervention was more effective in mitigating these alterations than individual strains. This protective effect parallels with the observations in bovine epithelial models, where *Lactobacillus* intervention alleviated oxidative injury by inhibiting ROS accumulation via the mitochondrial pathway and boosting antioxidant factors (Feng et al., 2025). Furthermore, the enhanced antioxidant capacity observed in the probiotic mixture (Bo et al., 2022) indicates that the treatment successfully alleviated the systemic pathological burden associated with colitis.

Epithelial defects may not only be a consequence of IBD, but may also be a contributing factor (Odenwald et al., 2016). Epithelial disruption, as the interface integrating microbial and immune signals, may disturb microbiota diversity and promote immune dysregulation associated with IBD (Martini et al., 2017). Given the central role of the barrier in disease pathogenesis, the administration of probiotics might enhance gut integrity and potentially exert disease-preventive benefits (Wong et al., 2022). In the present study, the combined probiotic intervention demonstrated the most potent restorative effect on goblet cells and the expression of *ZO-1* and *occludin*, significantly outperforming the efficacy of individual strain treatment. This suggests that the intestinal barrier has been restored. *P. acidilactici* demonstrates strong gastrointestinal tolerance and bacteriocin production, potentially establishing an early ecological niche and reducing gut pH via lactic acid, thereby facilitating the colonization of *L. acidophilus* (Parada et al., 2007; Li et al., 2021). *L. acidophilus* may reinforce epithelial integrity via strong adhesion and aggregation capacities (Goh and Klaenhammer, 2010). This probiotics cooperation likely strengthened the physical barrier, restricting the entry of luminal pro-oxidants and inflammatory stimuli into the circulation.

The improvements observed in host physiology are intrinsically linked to the restructuring of the gut microbiome. DSS-induced colitis is typically characterized by dysbiosis of gut microbiota. Reduced microbial diversity is a common feature noted in UC (Ohkusa and Koido, 2015). Interestingly, in contrast to the typical α-diversity decline observed in UC models, we found an increase in α-diversity. This increase is likely due to the expansion of pathogenic bacteria, which enhanced species richness while reducing functional stability. Consistently, DSS treatment induced obvious disruption of the Firmicutes/Bacteroidota (F/B) ratio, which is a hallmark of colitis-associated imbalance (Stojanov et al., 2020). The combined probiotic intervention more effectively reversed these structural shifts compared to individual strains, as evidenced by the restoration of the F/B balance and diversity normalization. It was noting that a specific restorative effect was observed in *Akkermansia* abundance in the present study. Together with report by others, DSS treatment reduced the relative abundance of *Akkermansia* (Derrien et al., 2010). Combined probiotics resulted in an increase of *Akkermansia* beyond those observed in both the DSS and NC groups. This is consistent with findings that *P. acidilactici*-containing mixtures can elevate the abundance of *Akkermansia* (Li et al., 2022). Consistent with this enrichment, *Akkermansia*, *Lachnospira,* and *Faecalibacterium* were, respectively, the key biomarkers in the T1, T2, and T3 groups. *Akkermansia* abundance showed a significantly positive correlation with butyrate concentration. By degrading host mucus, *Akkermansia* releases oligosaccharides that serve as fermentation substrates for butyrate-producing genera such as *Lachnospira* and *Faecalibacterium* (Lenoir et al., 2020; Nel Van Zyl et al., 2022). In turn, butyrate supports colonocyte energy metabolism and promotes mucin production, with mucins forming a protective mucus barrier and serving as substrates for *Akkermansia*, thus creating a positive feedback loop (Geirnaert et al., 2017; Chen and Vitetta, 2020).

The superior restorative effect observed in the combined group aligns with the established “cross-feeding” loop model between *Akkermansia* and butyrate (Rios-Covian et al., 2016; Belzer et al., 2017), While the interaction requires further causal validation, this framework offers a plausible mechanistic explanation for the superior therapeutic efficacy of the combined probiotics. Correlation analysis revealed that butyrate concentration was negatively correlated with the inflammation indexes and positively associated with antioxidant signatures and increase of tight junction proteins. Based on these, we hypothesize that the elevated butyrate levels might exert their protective effects through several well-documented pathways. For instance, butyrate is known to function as a histone deacetylase inhibitor, potentially suppressing the NF-κB and/or MAPK signaling pathways, which enhance the activity of antioxidant enzymes by inducing the Nrf2 pathway (Lee et al., 2017; Tang et al., 2022). Similarly, *Akkermansia* is classically characterized as a mucin-degrading genus linked to the restoration of barrier integrity (Jian et al., 2023). Previous studies have demonstrated that *Akkermansia*-derived extracellular vesicles can regulate intestinal permeability through the occludin pathway (Chelakkot et al., 2018a). Furthermore, *Akkermansia* may improve the inflammatory microenvironment by inhibiting Toll-like receptor (TLR) expression, which in turn suppresses NF-κB-mediated inflammation (Shen et al., 2023). It is important to acknowledge that the current study relies on correlational data; therefore, these results should be viewed as an interpretative model consistent with our findings. The current study suggests a potential mechanism that the combination of certain strains restore intestinal homeostasis by shaping a synergistic microbiome–metabolite network. This network subsequently strengthens the epithelial barrier, scavenges ROS, and dampens inflammatory signaling. Needless to say that future investigations such as microbial depletion models or functional interventions utilizing gene knockout models or specific inhibitors (e.g., those targeting TLR or Nrf2 signaling) are required to causally validate this cross-feeding loop and its subsequent impact on intracellular signaling.

We anticipate several drawbacks that may affect the validity of our findings. The preventive intervention model highlights the prophylactic potential of the combined probiotics; however, their therapeutic efficacy at the molecular and cellular levels requires further investigation. Moreover, the administration of non-encapsulated strains poses a challenge regarding bacterial viability during gastrointestinal transit, although the relatively high dosage used in the present study aimed to compensate for potential losses. Furthermore, testing of only two strains raise questions regarding the generalization of their interaction principles to other probiotic combinations. Nevertheless, the results of this study suggest that effective probiotic formulation relies on ecological and metabolic complementarity, rather than simple strain mixing of different strains. Future work should integrate microbial ecology and metabolite-based criteria to support mechanism-informed probiotic design.

## 5 Conclusion

This study demonstrates that the combined administration of *L. acidophilus* and *P. acidilactici* can effectively alleviate colitis in mice, as evidenced by improvements in clinical symptoms, colonic histopathology, and inflammatory status. The probiotic intervention was also associated with enhanced antioxidant capacity, modulation of tight junction protein expression, partial restoration of gut microbiota composition, and increased cecal butyrate levels. Furthermore, among the tested conditions, the 2:1 ratio of *L. acidophilus* to *P. acidilactici* exhibited the most pronounced therapeutic efficacy. These findings support the concept that selecting ecologically complementary strains is a promising strategy for probiotic combination. Future studies are warranted to further verify the translational potential of this specific combination.

## Data Availability

The 16S rRNA sequencing datasets presented in the study are publicly available. This data can be found here: NCBI BioProject, accession number PRJNA1371999 (https://www.ncbi.nlm.nih.gov/bioproject/PRJNA1371999/).
